# The Association between Dominant Macular Drusen and Central Retinal Artery Occlusion in Young Females with Cardiac Valve Disease

**DOI:** 10.4103/0974-9233.51989

**Published:** 2008

**Authors:** Hassan Al-Dhibi, Manal Bouhaimed

**Affiliations:** 1 From the Vitreoretinal Division, King Khaled Eye Specialist Hospital, Riyadh, Saudi Arabia; 2 From the Department of Surgery, Faculty of Medicine, Kuwait University, Kuwait

**Keywords:** dominant macular drusen, central artery occlusion, cardiac valve disease

## Abstract

To describe uncommon association between central retinal artery obstruction and dominant macular drusen in two young female patients. First patient, a 22-year-old female was presented with right central artery obstruction associated with bilateral dominant macular drusen. Systemic evaluation disclosed the presence of mitral valve regurge. Second patient, a 34-year-old female with a previous history of right central retinal artery obstruction diagnosed elsewhere. Fundus exam showed bilateral dominant macular drusen and her systemic evaluation revealed severe rheumatic valve stenosis, moderate aortic regurge with moderate to severe tricuspid regurge and she underwent mitral valve replacement. To the best of our knowledge, the association between central retinal artery obstruction and dominant macular drusen was not previously reported.

Occlusive disorders of the retinal circulation are among the most dramatic problems encountered by the ophthalmologists because of their rapid onset, their potentially profound effects on vision, and their strong association with life-threatening systemic diseases.^1^ The finding of central retinal occlusion (CRAO) generally merits a complete systemic & ocular work up to look for etiologic factors.

The causes of retinal arterial occlusion in young adult patients often differ from those found in older adults.^2^ Disease entities that more commonly cause retinal arterial obstructive disease in young include migraine, coagulation abnormalities, cardiac disorders, trauma, sickle cell hemoglobinopathies & ocular abnormalities such as optic nerve drusen,^3^ peripapillary arterial loops,^4^ increased intraocular pressure,^1^–^6^ tosoplasmas^7^ and, optic neuritis.^8^

We report herein, two cases of right central retinal artery occlusion associated with bilateral dominant macular drusen in young adult females with cardiac valve diseases.

## Case Report

### Case 1:

A 22-year-old female presented with sudden loss of vision in her right eye for 10 hours duration with past ocular history of intermittent visual obscurations in both eyes for the last 3 years. Details of ocular examination can be seen in Table 1.

**Table 1:** Ocular Examination of Case 1.

Right Eye	Left Eye
Visual acuity: Hand motion	20/20

Intra ocular pressure:21 mmHg	21 mmHg

Pupil: 3+ Afferent papillary defect	4mm

**Figures 1 and 2:**
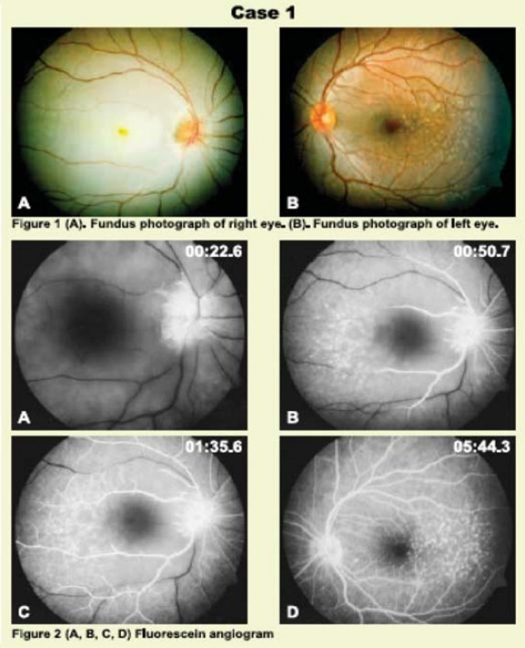
Fundus examination and fluorescein angiography of Case 1.
- Cardiac evaluation was performed by a cardiologist and her carotid doppler was within normal and echocardiography showed mild mitral regurge. The patient was treated with Aspirin.

Fundus Examination **(Figure 1)**:	Fundus Examination **(Figure 1)**:

1- Superficial whitening of the retina with cherry red spot.	1-Multiple temporal macular drusen with retinal pigment epithelial changes

2- Attenuated retinal blood vessels	

3- Multiple temporal macular drusen with retinal pigment epithelial changes	

Diagnosis: right central retinal artery occlusion; Management: immediate paracentesis, ocular massage in conjunctions with Carbogen and Diamox.

Investigation: The following were within normal limits:

- Physical examinations, CBC, ESR, Electrolytes, Hgb electrophoresis, PT, PTT, protein electrophoresis, central plasma viscosity, lipid profile, plasma homocysteine, blood sugar, C proteins, antiphospholipid antibodies, plasma fibrinogen, lipoprotein A, abnormal factor V leiden, antithrombin III deficiency, protein S and C deficiency.
- Intravenous fluorescein angiography **(Figure 2)** revealed a delay in retinal arterial filling with prolong arteriovenous transit time with ground glass appearance in the right eye and sharply demarcated hyper fluorescent spots in the temporal aspect of macular in both eyes.

### Case 2:

A 34-year-old female referred with diagnosis of right retinal arterial occlusion presented with history of decreased vision in her right eye for 1 year after an attack of cerebrovascular accident with left side weakness. Surgical and medical history revealed diagnosis of heart valvular disease and she underwent mitral valve replacement three years ago and using warfarin and digoxin. Details of ocular examination can be seen in Table 2.

**Table 2:** Ocular Examination of Case 2.

Right Eye	Left Eye
Visual acuity: 20/200	20/20

Intra ocular pressure:19 mmHg	19 mmHg

Pupil: 3+ Afferent papillary defect	4mm

**Figures 3 and 4:**
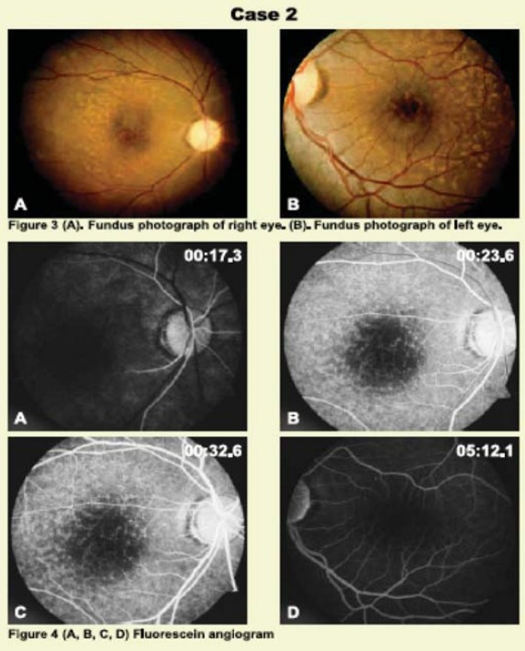
Fundus examination and fluorescein angiography of Case 2.

Fundus Examination **(Figure 3)**	Fundus Examination **(Figure 3)**

1- Pale disc, attenuated retinal blood vessels and no retinal neovascularizations	1- Macular drusen

2- Sclerosed macular arterioles with retinal pigment epithelial changes	

3- Macular drusen	

Diagnosis: old recanalized right central artery occlusions with bilateral macular drusen.

Investigation: Systemic evaluation by her Cardiologist showed rheumatic mitral valve stenosis, moderate aortic regurgitations with moderate to severe tricuspid regurge status post mitral valve replacement.

Intravenous fluorescein angiography **(Figure 4)** revealed to some extent a delay in arterial filling with prolong arteriovenous transit time in right eye and hyper fluorescent spots macular area of both eyes.

## Discussion

Dominant macular drusen (Doyne honey comb dystrophy) is an autosomal dominant disorder, which mimics drusen seen in age-related macular degeneration. This disorder occurs in younger patients at 20 to 30 years of age and the extent of the drusen is variable, with most cases limited to the posterior pole. Affected patients may later develop choroidal neovascularization. It may be relevant that drusen like deposits can be seen in some renal disorders that involve basement membrane abnormalities, such as Alport's syndrome and membranoproliferative glumerulonephritis Type II.^9^,^10^

Further literature review showed no reports of association of dominant macular drusen with other systemic disorders, particularly cardiac valvular disorders. Our cases revealed a trait of young adult females with bilateral dominat macular drusen, each of them presented with right central artery occlusion secondary to cardiac valvular disease. This association is based on observations which could be coincidental findings. And to the best of our knowledge these associations have not been reported previously.
